# Enhancing Cricket Performance Analysis with Human Pose Estimation and Machine Learning

**DOI:** 10.3390/s23156839

**Published:** 2023-08-01

**Authors:** Hafeez Ur Rehman Siddiqui, Faizan Younas, Furqan Rustam, Emmanuel Soriano Flores, Julién Brito Ballester, Isabel de la Torre Diez, Sandra Dudley, Imran Ashraf

**Affiliations:** 1 Institute of Computer Science, Khwaja Fareed University of Engineering and Information Technology, Abu Dhabi Road, Rahim Yar Khan 64200, Punjab, Pakistan; hafeez@kfueit.edu.pk (H.U.R.S.); younasfaizan97@gmail.com (F.Y.); 2 School of Computer Science, University College Dublin, D04 V1W8 Dublin, Ireland; furqan.rustam1@gmail.com; 3 Engineering Research & Innovation Group, Universidad Europea del Atlántico, Isabel Torres 21, 39011 Santander, Spain; emmanuel.soriano@uneatlantico.es (E.S.F.); julien.brito@uneatlantico.es (J.B.B.); 4 Department of Project Management, Universidad Internacional Iberoamericana Campeche, Campeche 24560, Mexico; 5 Department of Projects, Universidad Internacional Iberoamericana Arecibo, Puerto Rico, PR 00613, USA; 6 Project Management, Universidade Internacional do Cuanza, Cuito EN250, Angola; 7 Fundación Universitaria Internacional de Colombia Bogotá, Bogotá 11001, Colombia; 8 Department of Signal Theory, Communications and Telematics Engineering, University of Valladolid, Paseo de Belén, 15, 47011 Valladolid, Spain; 9 Bioengineering Research Centre, School of Engineering, London South Bank University, 103 Borough Road, London SE1 0AA, UK; dudleyms@lsbu.ac.uk; 10 Department of Information and Communication Engineering, Yeungnam University, Gyongsan-si 38541, Republic of Korea

**Keywords:** batsman stroke prediction, computer vision, machine learning, random forest

## Abstract

Cricket has a massive global following and is ranked as the second most popular sport globally, with an estimated 2.5 billion fans. Batting requires quick decisions based on ball speed, trajectory, fielder positions, etc. Recently, computer vision and machine learning techniques have gained attention as potential tools to predict cricket strokes played by batters. This study presents a cutting-edge approach to predicting batsman strokes using computer vision and machine learning. The study analyzes eight strokes: pull, cut, cover drive, straight drive, backfoot punch, on drive, flick, and sweep. The study uses the MediaPipe library to extract features from videos and several machine learning and deep learning algorithms, including random forest (RF), support vector machine, k-nearest neighbors, decision tree, linear regression, and long short-term memory to predict the strokes. The study achieves an outstanding accuracy of 99.77% using the RF algorithm, outperforming the other algorithms used in the study. The k-fold validation of the RF model is 95.0% with a standard deviation of 0.07, highlighting the potential of computer vision and machine learning techniques for predicting batsman strokes in cricket. The study’s results could help improve coaching techniques and enhance batsmen’s performance in cricket, ultimately improving the game’s overall quality.

## 1. Introduction

Human pose estimation (HPE) is a rapidly developing field of research that employs computer vision techniques to estimate the positions of various human body components in images or video footage. Despite recent advancements in computer vision, accurately understanding human actions from visual data is still challenging. Human body movements are often driven by unique activities, making identifying and categorizing them accurately difficult. Understanding a person’s body pose is crucial for identifying their actions, which is where HPE techniques come in handy. By recognizing and categorizing human body joints, such as the head, arms, and torso, HPE can capture coordinates for each joint that define a person’s position [1].

In sports analytics, computer vision has become increasingly crucial for extracting valuable insights from various forms of visual data [2]. Coaches and athletes can use computer vision techniques to track and analyze movement patterns during games or practice sessions, providing valuable performance feedback, identifying areas for improvement, and making strategic decisions [3]. Additionally, computer vision can be used for activity recognition, outcome prediction, and injury prevention. Using computer vision in sports can revolutionize how we analyze and train athletes, improving their performance and reducing the risk of injury.

Human pose estimation, in particular, is an exciting area of research within sports analytics. With advancements in camera technology and computer vision algorithms, tracking of athletes’ body movements during training and competition has become more accurate over time [4]. This technology has significant applications in sports performance analysis and injury prevention. Coaches and athletes can monitor progress, identify areas for improvement, and prevent potential injuries by tracking body movements. Human pose estimation can also provide insights into the biomechanics of athletic movements, helping coaches and trainers optimize training methods and improve performance. The application of human pose estimation in sports extends to various sports, including basketball, soccer, and volleyball, making it an area of growing interest among researchers exploring its potential for improving athletic performance and reducing the risk of injury.

Human pose estimation through computer vision has revolutionized how cricket strokes are analyzed and predicted. By scrutinizing batsmen’s body posture and movements during a game, coaches and analysts gain detailed insights into their batting techniques and strategies [5]. Computer vision techniques are used to detect the orientation of the bat and the position of the batsman’s body, enabling the identification of different types of strokes played by the batsman. This data analysis helps recognize a batsman’s strengths and weaknesses, empowering coaches and players to optimize their training and gameplay. Furthermore, the integration of machine learning algorithms enables the system to forecast the type of shot the batsman is likely to play based on their previous performances [6]. Such predictions are instrumental in helping bowlers anticipate the shot and modify their strategy accordingly. For instance, if the system forecasts that the batsman is likely to play a cover drive, the bowler may adjust their line and length to make it more difficult to play that shot. In conclusion, human pose estimation using computer vision in cricket has exhibited enormous potential in enhancing performance analysis and improving training methods. It enables coaches and players to make data-driven decisions, ultimately improving their chances of winning. For accurate stroke prediction, the use of machine learning methods holds significant importance. In this regard, this study adopts a machine-learning approach for batsmen’s stroke prediction. This research makes several significant contributions to the field:

- The study collects a comprehensive video dataset to classify different cricket strokes. In contrast to previous studies that only use image datasets and cover a maximum of five strokes, this study covers eight strokes, including ‘flick’, ‘back foot punch’, ‘pull’, ‘cut’, ‘cover drive’, ‘straight drive’, ‘on drive’, and ‘sweep’.
- A novel technique is employed to extract features from the video dataset. The MediaPipe library extracts seventeen critical points of the human body. Based on these key points, the batsman’s stroke is accurately classified.
- The study uses fine-tuned machine learning and deep learning models to classify the strokes based on the extracted feature dataset. Cross-validation is employed to validate the model’s performance, ensuring accurate results.
- This research provides a more comprehensive and accurate approach to classifying cricket strokes. The novel technique that extracts features from video datasets and utilizes state-of-the-art machine learning and deep learning models helps improve classification accuracy.

The organization of the study is as follows: Section 2 examines the relevant literature studies on pose estimation and stroke recognition. Section 3 analyzes the workflow of the proposed methodology. The video stroke dataset and the technique used for feature extraction are also described. Results and discussions are presented in Section 4, and Section 5 concludes this study.

## 2. Related Work

Machine learning models have witnessed a wide adoption in various fields like image processing [7,8,9], text analysis [10,11], education [12,13], medical data analysis [14], etc., and sports is no exception. As a result, several studies have been presented involving the use of machine learning techniques in sports [15,16,17].

Human pose estimation for predicting players’ performance in sports has been investigated recently, leading to several techniques and approaches in this field. A recent study [18] proposed a batsman shorts estimation model to identify four different strokes in cricket: glance, drive, block, and cut. The study utilized an image dataset of cricket strokes and extracted feature vectors from head, feet, bat, and hand positions to train several models, including a k-nearest neighbor, support vector machine, and convolutional neural network (CNN)/AlexNet. The AlexNet model achieved the highest accuracy of 74.33%.

Along the same directions, ref. [19] extracted 15 critical data points from an image dataset of different cricket strokes using MediaPipe. The dataset was used to develop a mobile application to help batsmen improve their accuracy. The random forest (RF) model achieved an F1 score of 87%. In another study [20], a dataset of 63 different backward and forward cricket strokes was collected and classified using a long short-memory (LSTM) network and bidirectional LSTM models. Both models achieved 100% accuracy. The authors used motion vectors and three-dimensional (3D) match recognition to classify eight angles of cricket strokes with high precision in [21].

Action recognition using deep learning was also applied to other sports like badminton, table tennis, and high jump. For instance, in a recent study ([22]), the ResNet-18, VGG-16, and GoogleNet models were used to classify badminton smashes. The ResNet-18 achieved a high accuracy of 97.51% and 98.66% on training and testing, respectively. On the Jeston Nano hardware, the GoogleNet model outperformed, achieving 83.04% and 97.0% accuracy on training and testing, respectively. In one study ([23]), a new approach was utilized to collect data on the footwork of badminton players. This study used a deep-learning method to extract two-dimensional (2D) and 3D coordinates of the players’ shoes. The model achieved an absolute positioning accuracy of 74%. These data provide valuable insights into the players’ movements, which can help improve their performance on the court.

Study [24] employed a novel technique to gather data for the classification of different strokes played in table tennis. The authors collected a video dataset of the primary 11 strokes of 14 professional table tennis players and utilized CNN and other machine learning models to classify the strokes. The CNN model achieved an impressive accuracy of 99.37%. Similarly, ref. [25] studied classifying different human actions using a custom CNN model. The authors created two datasets, the first consisting of 10 actions obtained using the Kinect v2 sensor and the second comprising seven subjects performing 20 other actions. The model achieved 97.23% accuracy on the Kinect dataset and 87.1% on the MRS dataset.

A 13-layered conventional neural network called ‘short net’ is presented in [26] to classify six different strokes. These strokes include ‘cut shot’, ‘straight drive’, ‘cover drive’, ‘pull shot’, ‘leg glance shot’, and ‘scoop shot’. The model achieved good accuracy with a minimum entropy score. All the previous work on cricket stroke recognization is summarized in Table 1. Table 1 shows the dataset used in the previous studies, the stroke they classified, and the outperforming model with the reported accuracy.

## 3. Proposed Methodology

**Figure 1:**
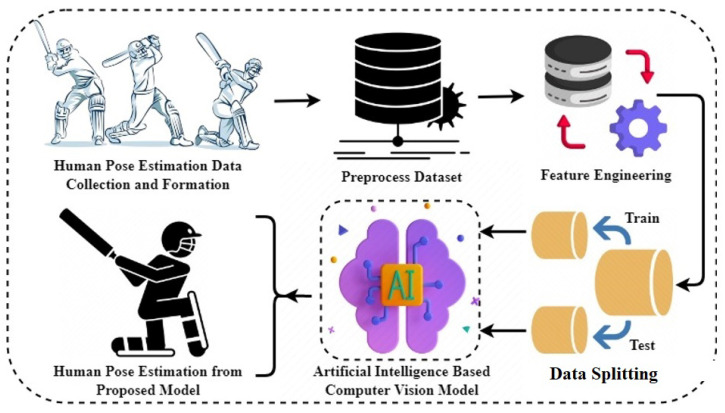
Workflow of adopted methodology.

The workflow of the proposed approach is presented in Figure 1. The study collected videos of eight different types of batsman strokes. The videos were preprocessed to remove any noise present to ensure accurate analysis. The MediaPipe library was used to extract human key points from the preprocessed videos, and a novel dataset was created based on these features. The dataset was preprocessed again to eliminate any remaining noise, and the analysis focused on 17 critical points of human movement. Before implementing machine learning and deep learning models on the dataset, it was split into test and train sets. The research dataset was used to train and test the models, and a performance evaluation was conducted to assess their effectiveness in real-time.

### 3.1. Video Cricket Strokes Dataset

**Figure 2:**
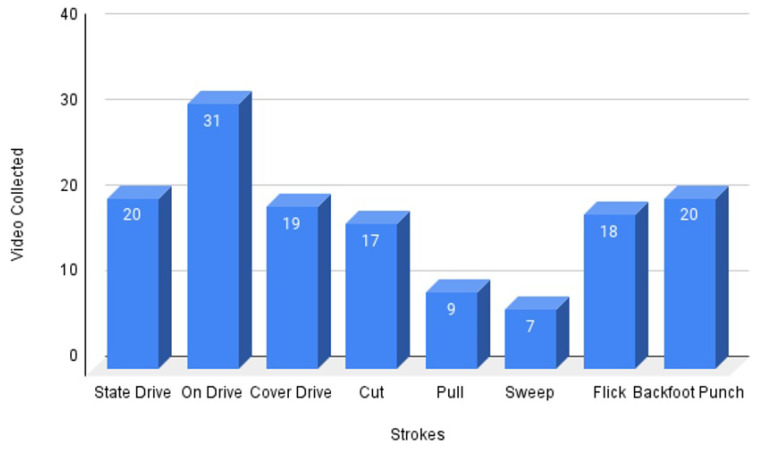
Number of videos for different strokes.

This study aims to create a comprehensive dataset of cricket stroke videos by collecting a diverse range of videos from various platforms. To ensure the dataset’s generalizability, the videos were collected from both YouTube channels and the Liaquat Pur cricket club, Pakistan, focusing on eight primary strokes: ‘pull’, ‘cut’, ‘cover drive’, ‘straight drive’, ‘backfoot punch’, ‘on drive’, ‘flick’, and ‘sweep’. Multiple videos of each stroke were collected to provide a diverse range of examples for analysis. The count plot in Figure 2 visually represents the number of videos collected for each stroke. The *x*-axis displays the number of videos, and the *y*-axis displays the type of strokes. This information provides an overview of the distribution of videos in the dataset.

**Figure 3:**
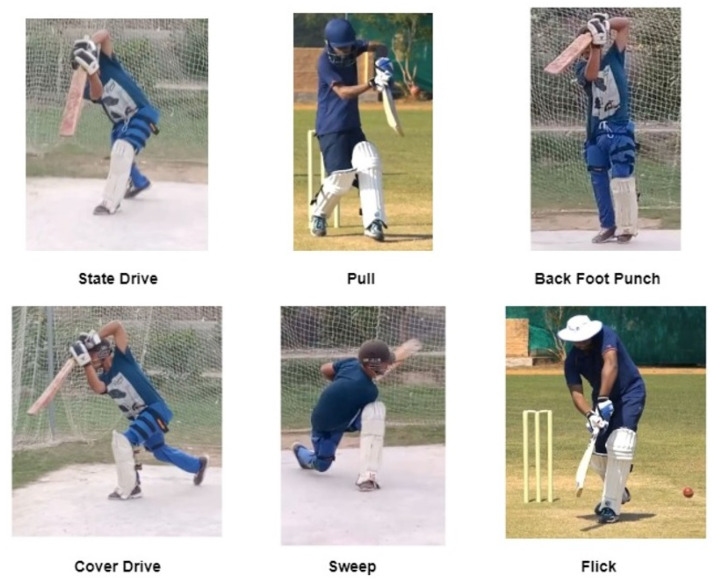
Sample frames from different videos.

To ensure accurate analysis, the recorded video data were preprocessed, and any noise present was manually removed. Each stroke video has a length of approximately 1.5 to 2 s, providing a consistent length for analysis. A few sample frames from the recorded videos are shown in Figure 3, demonstrating the video quality. The resulting dataset provides a valuable resource for researchers to analyze and compare different cricket stroke techniques. The diverse range of videos ensures that the dataset is comprehensive and can be used to study the nuances of each stroke.

### 3.2. Feature Extraction from Videos

Once the cricket stroke videos are preprocessed and the noise removed, the MediaPipe library extracts features from the videos. This library is a pre-built set of components that can be used to create complex machine-learning models for tasks such as pose estimation, facial recognition, hand tracking, and object detection. It can extract 33 landmarks from the human body pose estimation. The pose landmarks *P* can be used to represent the pose of a person in various ways. One common representation is the skeletal representation, where the pose landmarks are connected by lines to form a skeletal structure representing the person’s body. The skeletal representation can be represented as follows:

(1) $$S=\{l_{i}\}$$

where

(2) $$l_{i}=\left(p_{i},p_{j}\right),i,j\in \left\{1,2,\ldots ,17\right\},i<j$$

The value *S* is the set of 16 lines that connect the 33 pose landmarks *P* to form the skeletal structure, and $p_{i}$ and $p_{j}$ are the two endpoints of the *i*th line. The pose estimation pipeline can be summarized as follows:

(3) I→f→P→S

where *I* is the input frame from the video, *f* is the deep neural network that performs the pose landmark estimation, *P* is the set of 33 pose landmarks, and *S* is the skeletal representation of the pose.

For this study, only 17 landmarks were selected, as they are critical to detecting strokes, namely the nose, left shoulder, right shoulder, left elbow, right elbow, left wrist, right wrist, left hip, right hip, left knee, right knee, left ankle, right ankle, right heel, left heel, left foot index, and right foot index. The MediaPipe library extracts the 17 landmark points and their *x*, *y*, and *z* coordinate values from every video frame.

The MediaPipe library also provides a visibility value that can be set to extract features from the videos. This study’s visibility value was set to extract 17 landmarks only. The OpenCV library passes every video to the MediaPipe library to extract the landmark points. Extracting these landmarks creates a new data frame containing 51 feature columns and one label column named as cricket stroke dataset. The working of the proposed approach is shown in Algorithm 1.
**Algorithm** **1** Batsmen stroke prediction.**Input:** Video strokes dataset (VSD)**Output:** Stroke prediction {cover driver, pull, sweep, state drive, on drive, cut and back foot punch}
1:$MP_{F}\leftarrow$ MediaPipe(VSD) // VSD ∈ Video strokes dataset, MPF ∈ extracted features from the MediaPipe library.2:$T_{RF}\leftarrow RF_{training}\left(MP_{F}T_{e}\right)$ // $MP_{F}T_{e}$∈MPF, here $MP_{F}T_{e}$ is the training data of MPF.3:$RF_{Pred}\leftarrow T_{RF}\left(MP_{F}T_{s}\right)$ // $MP_{F}T_{s}\in MPF$, here $MP_{F}T_{s}$ is the testing data of MPF, $RF_{Pred}\in${cover drive, pull, sweep, state drive, on drive, cut and back foot punch}


### 3.3. Cricket Stroke Exploratory Data Analysis

This section deeply explores the cricket stroke dataset after extracting features from the videos. The new dataset contains 51 feature columns. The 51 feature columns correspond to the *x*, *y*, and *z* coordinates of each of the 17 selected landmark points. The label column contains the name of the stroke performed in the video. The column names for the features and labels are shown in Table 2.

The cricket strokes dataset (CSD) is a collection of numeric features extracted from videos using the MediaPipe library, resulting in 8998 records. However, the final dataset is not balanced, with different strokes having varying numbers of instances. Specifically, the dataset includes 1060 records for ‘straight drive’, 2276 instances for ‘on drive’, 1236 records for ‘cover drive’, 1011 rows for ‘cut’, 779 records for ‘pull’, 511 records for ‘sweep’, 908 instances for ‘flick’, and 1217 records for ‘back foot punch’. A summary of the dataset is presented in Table 3. The highest percentage belongs to ‘on drive’ with 25.29% instances, whereas ‘sweep’ has the lowest ratio to the total records at 5.68% of the total records.

**Figure 4:**
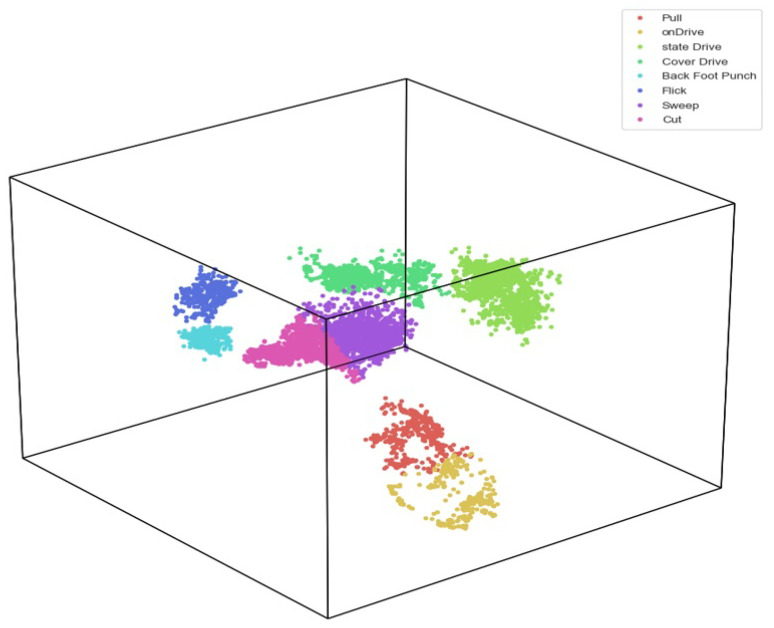
Feature space analysis.

The cricket strokes dataset is analyzed in three-dimensional space. A Python library HyperTools created a cubic scatter plot. HyperTools uses dimensionality reduction to visualize high-dimensional data in a lower-dimensional space using the t-distributed stochastic neighbor embedding (t-SNE) technique. The features extracted from the video are more detachable, and the machine learning model can easily classify these features, as shown in Figure 4.

**Figure 5:**
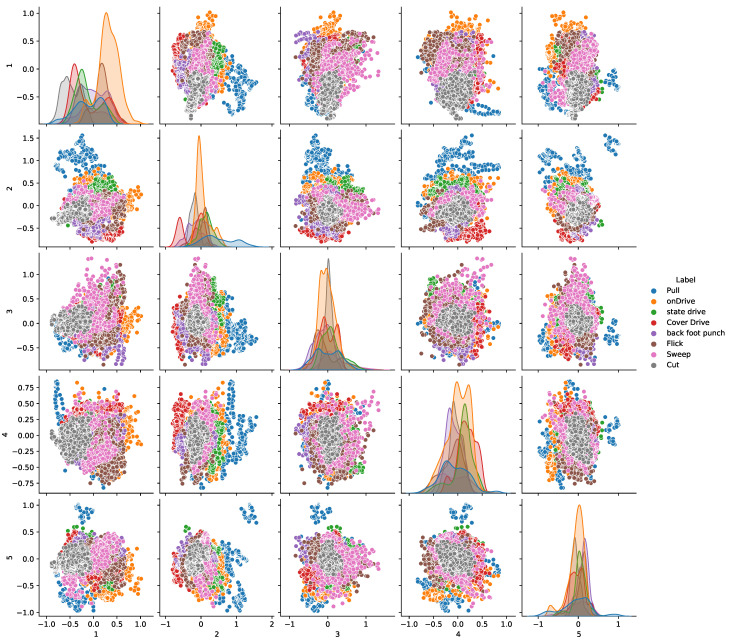
Pair plot on extracted features.

The pair plot is plotted on the dataset to check the correlation between different features. We extract the five most important features from the dataset using principal component analysis to plot the pair plot on these features. The pair plot shows that these points are more easily detachable, as shown in Figure 5.

### 3.4. Target Label Encoding

Label encoding is a common technique used in machine learning to convert categorical variables into numerical representations. Label encoding is necessary because many machine learning algorithms require input data to be in numerical format. Label encoding assigns a unique numerical value to each category within a variable, allowing the algorithm to identify patterns and relationships within the data. The label column is encoded in this study, and every class is assigned a different serial number from 0 to 7.

### 3.5. Dataset Splitting

Dataset splitting is a technique used in machine learning to partition a dataset into two subsets: a training dataset, and a testing dataset. The purpose of this is to assess the performance of a machine learning model on unseen data, which can help to identify whether the model is overfitting, underfitting, or generalized. This study splits the dataset into three different ratios, 70:30, 80:20, and 90:10, and gets the accuracy on all splits. On the 80:20 data split, the models give high accuracy.

### 3.6. Model Training

Various machine learning and deep learning models were applied to the cricket strokes dataset to classify the batsmen’s strokes. The machine learning models used in the study included LSTM, k-nearest neighbor (KNN), logistic regression (LR), decision tree (DT), support vector machine (SVM), and RF. A hyperparameter tuning process was applied to these models to obtain optimal results. The specific parameters used for the machine learning models are outlined in Table 4.

### 3.7. Performance Metrics

Several performance matrices are used in this study to evaluate the performance of machine learning algorithms. Precision, recall, and F1 score are three standard evaluation metrics in machine learning classification tasks. With these, standard evaluation matrix geometric mean, Cohen’s kappa, and log loss are also measured to evaluate the performance of machine learning models.

## 4. Results and Discussion

This section discusses the results of the applied machine learning and deep learning models. Various performance metrics, such as precision, recall, F1 score, Cohen’s kappa, geometric mean, and log loss were used to evaluate the models’ performance. Different machine learning and deep learning models are applied to the CSD dataset. A hyperparameter tuning technique was applied to each model to achieve optimal results. The parameters used in the machine learning models are explained in Table 4.

### 4.1. Results for Machine and Deep Learning Models

All the performance measures are evaluated on three different data splits, including 70:30, 80:20, and 90:10 for training and testing, respectively. The deep learning-based LSTM model has the lowest precision, recall, and F1 score value, as shown in Table 5. The RF has the highest value of precision, recall, and F1 score on all the data splits in comparison with all the other machine learning models. RF achieves the highest precision, recall, and F1 score on an 80:20 data split.

On other performance measures including the Cohen kappa score and geometric mean, the RF model also outperforms all the employed models. On the 80:20 data split, the RF has the highest value of Cohen kappa and geometric mean score. LSTM has the lowest value of Cohen kappa and geometric mean score on every data split. The LSTM model has the highest log loss value compared to all other applied machine learning models. RF has a 0.076 log loss on an 80:20 split, as shown in Table 6.

The performance of several machine learning and deep learning models was evaluated on the cricket strokes dataset. Precision, recall, F1 score, Cohen’s kappa, geometric mean, and log loss were used as performance metrics to measure the effectiveness of the models. The models were trained on three different data splits to ensure the robustness and generalizability of the results. The results showed that the deep learning-based LSTM model had the lowest accuracy compared to all other machine learning models. The RF model outperformed all other models in terms of accuracy on all three data splits, as shown in Table 6.

**Figure 6:**
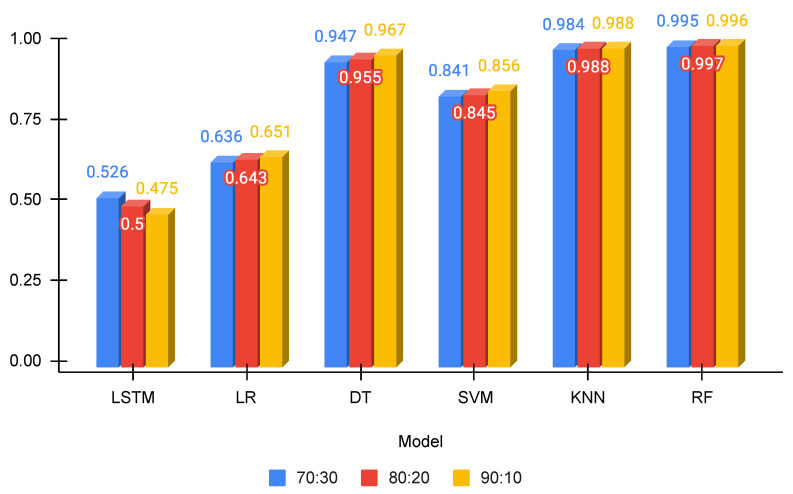
Accuracy scores for all models.

Figure 6 shows the visual presentation of models’ accuracy for three training and testing data splits. It can be observed that the accuracy of DT, KNN, and RF is higher than the 0.94 score, whereas LSTM and LR show poor performance. The performance of SVM is moderate with a 0.841 accuracy score. However, the best performance is obtained by the RF, which has a 0.997 accuracy score.

**Figure 7:**
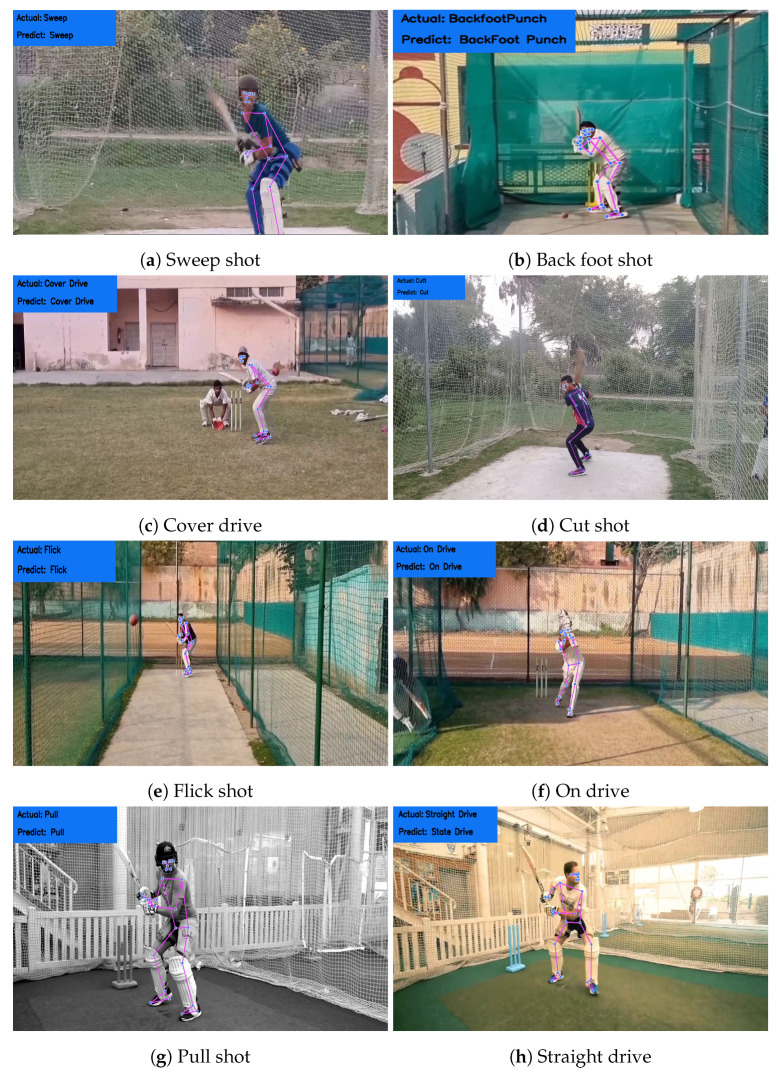
Sample predictions from the RF model.

The RF model has the highest accuracy on the 80:20 data split, as illustrated in Figure 7. Furthermore, the RF model has the lowest time complexity on the 80:20 data split, indicating that it could classify cricket strokes more efficiently. Based on these results, it can be concluded that the RF model is the best machine-learning model for classifying cricket strokes.

### 4.2. K-Fold Cross-Validation Analysis

Cross-validation is a crucial statistical technique used in machine learning to mitigate the effects of overfitting and enhance the model’s generalization performance. Overfitting is a common problem in machine learning where a model is too complex and fits the training data too closely, leading to poor performance on new or unseen data. To overcome this problem, cross-validation involves dividing a dataset into subsets, where one subset is used as the validation set to test the model’s performance while the remaining subsets are used for training the model. This process is repeated multiple times, each subset taking turns as the validation set. The results of each iteration are averaged to provide an estimate of the model’s performance.

Using cross-validation, a model’s ability to generalize to new data can be evaluated more accurately. It helps to identify models that are overfitting the training data and allows for selecting the best-performing model. In this study, the researchers used a 60-fold cross-validation to evaluate the performance of the random forest model. In K-fold cross-validation, the dataset is divided into *K* equally sized folds, and the model is trained and validated *K* times.

In this study, *K* was set to 60, which provides a more reliable estimate of the model’s performance than a smaller value of *K*. The cross-validation results indicate that the RF model achieves an accuracy of 95% with a standard deviation of 0.07. This high accuracy and low standard deviation indicate that the model performs well and is consistent across different folds.

Overall, cross-validation is a powerful technique for evaluating the performance of machine learning models. It helps mitigate the overfitting effects and enhances the model’s generalization performance. The use of 60-fold cross-validation in this study provides a more accurate estimate of the RF model’s performance and enables the selection of the best-performing model, as shown in Table 7.

### 4.3. Time Complexity

The time complexity of a machine learning model is a critical factor that refers to the amount of computational resources required to train and test the model on a given dataset. The time complexity can vary for different machine learning models and can be influenced by various factors, such as the size and complexity of the dataset, the number of features, and the model architecture.

The time complexity of a machine learning model is an essential consideration for several reasons. First, it can affect the model’s scalability, mainly when dealing with large and complex datasets. Second, it can impact the speed and efficiency of the model, which can be critical when dealing with real-time or near-real-time applications. Finally, it can influence the cost and feasibility of deploying the model in production environments.

This study measured the time complexity of different machine learning models on different data splits, as shown in Table 8. The time computation was measured in seconds, and the results indicate that the KNN model has the lowest time computation on the 70:30 data split. In contrast, the RF model has the lowest time complexity on the 80:20 data split, where it also provides the best results for the same training–testing split.

### 4.4. Performance Comparison

Performance comparison with existing studies was also carried out in this study. For this purpose, models from existing studies for batsmen’s stroke prediction are selected. The studies [18,19,20] use images and video datasets to predict different types of strokes. These studies employ AlexNet, LSTM, and RF models for stroke prediction. Performance analysis given in Table 9 indicates that the RF used in the current study shows better performance with a higher number of strokes prediction and outperforms existing approaches.

### 4.5. Discussion

The use of human pose estimation holds several strategic advantages in sports. It can be used by coaches to train players better and enhance their sports performance. Cricket, being the second most popular sport in the world, is liked and followed by billions of people around the globe. Consequently, coaches and players are continuously striving for excellence. The use of machine learning techniques to predict batsmen’s strokes can be very influential and useful in this regard. This study collects video data for different strokes and proposes a machine-learning approach for stroke prediction. Different important features are extracted from the preprocessed video data to train machine learning models. After training and testing the models, it was found that the RF model achieved the highest accuracy among all the other machine learning and deep learning models. The RF model has a higher precision, recall, F1 score, Cohen’s kappa, and geometric mean than other models. Additionally, the log loss was lower for the RF model. Overall, the results indicate that the RF model is the most suitable model for classifying the batsmen’s strokes in the CSD dataset. It can accurately identify the various types of strokes played by the batsmen with high precision and recall, making it a reliable tool for stroke analysis.

## 5. Conclusions

Cricket stroke classification is proposed in this study using a machine learning approach to enhance the performance of batsmen. Several machine learning and deep learning algorithms are used to benchmark the newly collected video dataset. A novel video dataset is created that contains eight types of strokes from cricket batsmen. The proposed approach is implemented along with other machine learning models to analyze its efficacy. Experimental results demonstrate that the proposed approach outperforms other models employed in this study. The RF model achieves a 0.997 accuracy, while the accuracy based on k-fold cross-validation amounts to 0.95 on an 80:20 data split. The proposed model achieves the highest accuracy and classifies the eight cricket strokes on a video dataset. This study demonstrates the significant impact of emerging technologies like computer vision and machine learning on sports. As these technologies advance, we can expect even more sophisticated and accurate predictions of batsman strokes and other critical aspects of cricket. In the future, more strokes will be added to the video dataset. We also intend to incorporate more features into the training process, such as angel measurement, acceleration, etc., to further improve the accuracy.

## Figures and Tables

**Table 1:** Summary of the literature review on cricket stroke prediction.

Refs.	Dataset	Strokes	Technique	Accuracy
[18]	Images	Glance, drive, block, and cut	AlexNet	74.33%
[19]	Images	Cut, cover drive, straight drive, pull, leg glance, scoop	Random forest	87%
[20]	Videos	Backward and forward	LSTM	100%
[18]	Videos	Strokes and gameplay	AlexNet	96.66

**Table 2:** Features in the dataset.

Attribute	Dtype	Attribute	Dtype	Attribute	Dtype
nosex	float	lshoulderx	float64	nosey	float64
lshouldery	float64	nosez	float64	lshoulderz	float64
rshoulderx	float64	lelbowx	float64	rshouldery	float64
lelbowy	float64	rshoulderz	float64	lelbowz	float64
relbowx	float64	rWristx	float64	relbowy	float64
rWristy	float64	relbowz	float64	rWristz	float64
lWristx	float64	rhipx	float64	lWristy	float64
rhipy	float64	lWristz	float64	rhipz	float64
lhipx	float64	rkneex	float64	lhipy	float64
rkneey	float64	lhipz	float64	rkneez	float64
lkneex	float64	rankelx	float64	lkneey	float64
rankely	float64	lkneez	float64	rankelz	float64
lankelx	float64	rheelx	float64	lankely	float64
rheely	float64	lankelz	float64	rheelz	float64
lheelx	float64	lfindexx	float64	lheely	float64
lfindexy	float64	lheelz	float64	lfindexz	float64
rfindexx	float64	rfindexy	float64	rfindexz	float64

**Table 3:** Number of instances of every stroke.

Strokes	Records	Percentage
State Drive	1060	11.78%
On Drive	2276	25.29%
Cover Drive	1236	13.74%
Cut	1011	11.24%
Pull	779	8.66%
Sweep	511	5.68%
Flick	908	10.09%
Backfoot Punch	1217	13.53%

**Table 4:** Hyperparameter settings for machine learning models.

Model	Hyperparameters
LSTM	loss = ‘categorical_crossentropy’, optimizer = ‘adam’, metrics = ‘accuracy’, activation=‘softmax’, batch_size = 64, validation_split = 0.1, epoch = 10
KNN	n_neighbors = 2
LR	C = 0.1, intercept_scaling = 10, random_state = 100
DT	random_state = 0, max_depth = 300
SVM	decision_function_shape = ‘ovo’, probability = True
RF	random_state = 0, max_depth = 300

**Table 5:** Experimental results for machine and deep learning models.

Model	Precision			Recall			F1 Score		
	70:30	80:20	90:10	70:30	80:20	90:10	70:30	80:20	90:10
LSTM	0.409	0.452	0.470	0.476	0.502	0.527	0.476	0.502	0.527
LR	0.658	0.655	0.649	0.637	0.643	0.651	0.637	0.643	0.651
DT	0.948	0.951	0.970	0.947	0.956	0.968	0.947	0.956	0.968
SVM	0.863	0.867	0.873	0.842	0.846	0.857	0.842	0.846	0.857
KNN	0.982	0.989	0.988	0.984	0.989	0.989	0.984	0.989	0.989
RF	0.996	0.998	0.996	0.995	0.998	0.997	0.995	0.998	0.997

**Table 6:** Cohen Kappa, geometric mean, and log loss for all models.

Method	Cohen Kappa Score			Geometric Mean Score			Log Loss			Accuracy		
	70:30	80:20	90:10	70:30	80:20	90:10	70:30	80:20	90:10	70:30	80:20	90:10
LSTM	0.372	0.413	0.431	0.526	0.50	0.475	0.0	0.0	0.0	14.09	13.26	12.82
LR	0.562	0.570	0.584	0.636	0.643	0.651	0.452	0.457	0.465	1.09	1.089	1.067
DT	0.938	0.947	0.962	0.947	0.955	0.967	0.941	0.955	0.969	1.895	1.601	1.161
SVM	0.812	0.817	0.831	0.841	0.845	0.856	0.823	0.829	0.848	0.449	0.438	0.420
KNN	0.981	0.986	0.987	0.984	0.988	0.988	0.983	0.988	0.990	0.358	0.231	1.709
RF	0.994	0.997	0.996	0.995	0.997	0.996	0.994	0.997	0.997	0.086	0.076	0.06

**Table 7:** Results for k-fold cross-validation.

Model	K-Fold Accuracy	Standard Deviation (±)
LSTM	0.115	0.172
LR	0.63	0.12
DT	0.87	0.10
SVM	0.83	0.13
KNN	0.94	0.06
RF	0.95	0.07

**Table 8:** Time complexity of all models for different training-testing splits.

Model	Time Computation (s)		
	70:30	80:20	90:10
LSTM	43.807	84.171	90.34
LR	0.914	0.518	0.649
DT	0.542	0.453	0.776
SVM	8.571	11.14	13.35
KNN	0.008	0.012	0.014
RF	7.542	5.174	5.695

**Table 9:** Performance comparison with existing approaches for batsmen stroke prediction.

Refs.	Strokes	Strokes	Model	Accuracy
[18]	Glance, drive, block, and cut	4	AlexNet	74.33%
[19]	Cut, cover drive, straight drive, pull, leg glance, scoop	6	RF	87%
[20]	Backward and forward	2	LSTM	100%
[18]	Strokes and gameplay	2	AlexNet	96.66%
This study	Straight drive, on drive, cover driver, cut, pull, sweep, flick, back foot punch	8	RF	99.7%

## Data Availability

Not applicable.
